# Control of plastid inheritance by environmental and genetic factors

**DOI:** 10.1038/s41477-022-01323-7

**Published:** 2023-01-16

**Authors:** Kin Pan Chung, Enrique Gonzalez-Duran, Stephanie Ruf, Pierre Endries, Ralph Bock

**Affiliations:** 1 Max-Planck-Institut für Molekulare Pflanzenphysiologie, Potsdam-Golm, Germany; 2 Universität Hamburg, Institut für Pflanzenwissenschaften und Mikrobiologie, Hamburg, Germany

**Keywords:** Plant genetics, Chloroplasts, Plant evolution, Genetic variation, Plant genetics

## Abstract

The genomes of cytoplasmic organelles (mitochondria and plastids) are maternally inherited in most eukaryotes, thus excluding organellar genomes from the benefits of sexual reproduction and recombination. The mechanisms underlying maternal inheritance are largely unknown. Here we demonstrate that two independently acting mechanisms ensure maternal inheritance of the plastid (chloroplast) genome. Conducting large-scale genetic screens for paternal plastid transmission, we discovered that mild chilling stress during male gametogenesis leads to increased entry of paternal plastids into sperm cells and strongly increased paternal plastid transmission. We further show that the inheritance of paternal plastid genomes is controlled by the activity of a genome-degrading exonuclease during pollen maturation. Our data reveal that (1) maternal inheritance breaks down under specific environmental conditions, (2) an organelle exclusion mechanism and a genome degradation mechanism act in concert to prevent paternal transmission of plastid genes and (3) plastid inheritance is determined by complex gene–environment interactions.

## Abstract (web summary)

Mitochondria and plastids are usually inherited maternally. Using genetic screens in tobacco, this study shows that low temperatures and knockout of the organellar exonuclease DPD1 lead to paternal transmission of plastids at high frequencies.

## Main

Cytoplasmic genomes are maternally inherited in most eukaryotes^1,2^. It is generally believed that the uniparental inheritance of organelles and their genomes makes them asexually reproducing genetic systems^3–5^. Lack of sexual recombination is expected to lead to the eventual mutational meltdown of organellar genomes, a phenomenon widely known as Muller’s ratchet^6–8^. This is due to the accumulation of deleterious mutations that cannot be separated from (only rarely occurring) beneficial mutations and can be considered as a case of ‘genetic hitchhiking’^9^. While there must be evolutionary forces that explain the strong prevalence of uniparental inheritance, there must also be compensatory mechanisms that allow organellar genomes to escape mutational meltdown.

In plants, the two organellar genomes (plastids and mitochondria) have lower mutation rates than nuclear genomes^10,11^. Low mutation rates reduce genetic hitchhiking and thus slow down Muller’s ratchet^8^. Recently, early mutation surveillance and induction of recombination repair (dependent on the MSH1 protein) has been proposed as part of a mechanistic explanation for the low mutation rates in plant organelles^12^. However, while low mutation rates can slow down Muller’s ratchet, they cannot entirely stop it from turning. Also, there are exceptional plant taxa, such as *Plantago, Silene* and *Pelargonium*, where organellar genome mutation rates are strongly elevated^13–16^. Together, these observations and considerations suggest that additional mechanisms are probably needed to prevent mutational meltdown in plants.

Besides low mutation rates, episodes of biparental inheritance of organelles could also potentially counteract Muller’s ratchet. Biparental transmission of organelles that can fuse (such as plant mitochondria) would allow them to participate in sex and recombination, thus providing the ability to generate genomes with favourable combinations of mutations and reduce genetic hitchhiking. Although fusion of seed plant plastids is only rarely observed^17–19^, biparental inheritance could help to resolve cytonuclear conflicts^20^, enable plastid genome capture^21,22^ and contribute to adaptive evolution^23,24^.

Although maternal inheritance is the general rule, stable biparental inheritance has arisen several times independently in the evolution of both animals and plants^25–29^. For example, the seed plants *Medicago* and *Pelargonium* show frequent biparental transmission^27,30^. Furthermore, even in plant species with largely maternal inheritance such as tobacco (*Nicotiana tabacum*) and *Arabidopsis thaliana*, the occasional transmission of plastids through pollen (‘paternal leakage’) has been documented, albeit at very low frequencies^31,32^. The reasons and the selective forces that have shaped uniparental organelle inheritance (while still allowing occasional paternal leakage), as well as the forces driving the evolutionary switches in the mode of organellar inheritance, are completely unknown.

Several cytological mechanisms that contribute to maternal inheritance of plastids have been described on the basis of microscopic observations. These include (1) exclusion of plastids from the generative cell in pollen mitosis I (PMI), (2) degradation of plastids and/or their DNA during pollen maturation and (3) elimination of paternal plastids during fertilization^33,34^. The molecular mechanisms involved in these processes are not well understood. In *Drosophila*, a nuclease (endonuclease G) degrades the paternal mitochondrial DNA upon fertilization^35^. In plants, two unidentified nuclear loci were associated with plastid inheritance patterns in *Pelargonium*^36^ but so far, not a single gene involved in determining the mode of cytoplasmic inheritance in seed plants has been identified. Also, the possible impact of environmental factors on the mode of plastid inheritance has not been explored.

As biparental inheritance is expected to profoundly affect organellar genome stability and evolution, we decided to study the determinants of cytoplasmic inheritance. To this end, we set out to identify environmental and genetic factors that determine plastid inheritance in the model plant tobacco.

## Results

### Screens for environmental influence on plastid inheritance

**Fig. 1:**
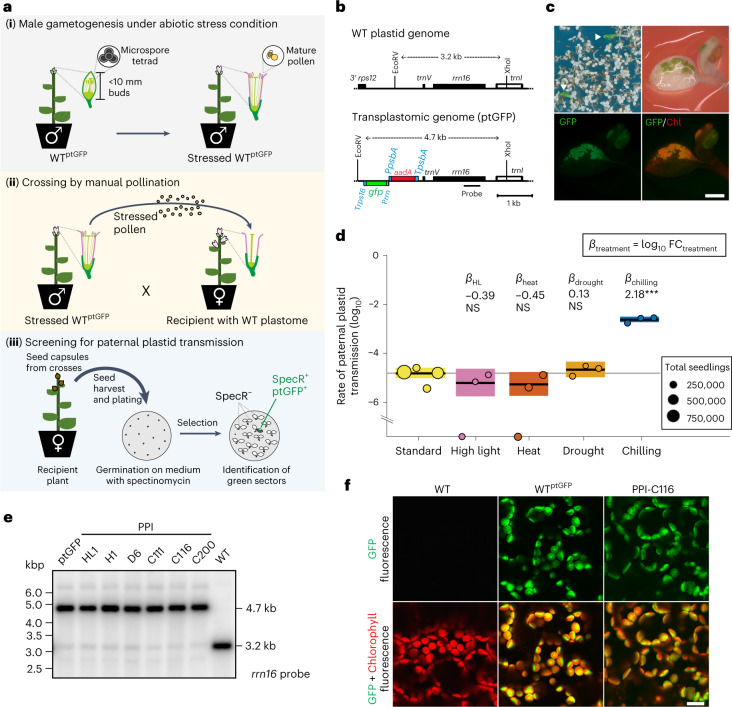
Identification of abiotic factors controlling plastid inheritance. **a**, Genetic screen for paternal plastid transmission. (**i**) At the onset of flowering, transplastomic plants (WT^ptGFP^) are exposed to abiotic stress so that the male gametophyte develops under stress. (**ii**) Greenhouse-grown plants with wild-type plastids are fertilized with pollen from stressed WT^ptGFP^ plants. (**iii**) Seeds are sown on spectinomycin-containing medium. Seedlings that inherited paternal plastids display green (spectinomycin-resistant) sectors^31^. **b**, Physical maps of the maternal (wild-type, WT) and paternal (transplastomic, ptGFP) plastid genomes. The paternal plastid genome harbours two transgenes: *aadA* (resistance marker) and *gfp* (reporter). Promoters, terminators (both blue) and relevant restriction sites are indicated. The black bar depicts a hybridization probe for RFLP. **c**, Paternal plastid transmission detected by spectinomycin selection. Top left: arrowheads indicate seedlings with green sectors. Top right: enlarged image of a green sector. Bottom: seedlings with green sectors displaying both GFP (left) and chlorophyll (Chl, right) fluorescence. Scale bar, 1 mm. **d**, Rates of paternal plastid transmission under stress. Circles represent proportions of seedlings carrying green, GFP-positive sectors per harvest (unit of replication, see Methods); circles in the *x* axis mean paternal transmission was not found. Transmission rates of stressed and untreated plants^31^ were compared, representing ‘Experiment 1’ (Table 1). Treatment effects (*β*) were estimated using Model 1 (*n*_rep.total_ = 16 harvests, ~4.35 million seedlings; Extended Data Tables 1 and 2) and tested by simultaneous two-tailed Wald *z*-tests. *α* = 0.05; NS, *P* > 0.05, ***P < 0.001. Only the chilling treatment has a significant effect (*P* = 1.22 × 10^−101^). *β* values represent fold changes in log_10_. Means per treatment are shown in black horizontal lines, with CI95 in coloured boxes. **e**, RFLP analysis of selected PPI lines: HL1, high light; H1, heat; D6, drought; C111, C116, C200, chilling. RFLP analysis with EcoRV and XhoI (cf. panel **b**) produces fragments of 4.7 kb for paternal plastids and 3.2 kb for maternal (WT) plastids. The blot is representative of three independent experiments. **f**, Localization of GFP fluorescence to chloroplasts. GFP fluorescence and the overlay with Chl fluorescence is shown for WT, transplastomic WT^ptGFP^ and a PPI line. Images are representative of a hundred independent PPI lines analysed. Scale bar, 10 µm. Source data

To analyse the effect of environmental factors on organellar genome inheritance, we employed a sensitive genetic screening procedure to detect and quantify events of paternal plastid entry into the zygote (Fig. 1a). Screening is based on incorporation of the antibiotic resistance gene *aadA* (conferring resistance to spectinomycin) and the reporter gene *gfp* (encoding the green fluorescent protein, GFP) into the plastid genome of the model plant tobacco (*Nicotiana tabacum*^31,37^; Fig. 1b). Spectinomycin is a specific inhibitor of protein biosynthesis in the chloroplast and its application to wild-type seedlings results in growth arrest and pigment loss. Biparental plastid transmission can be detected by pollinating wild-type plants (as maternal recipients) with pollen from plants containing the *aadA* marker gene in their plastid genome (as transplastomic father). Entry of paternal plastids into the zygote can be readily visualized by germinating seeds in the presence of spectinomycin because cells that receive paternal plastid genomes are resistant to the drug and remain green, thus resulting in green-white variegated seedlings (Fig. 1a,c).

**Extended Data Fig. 1:**
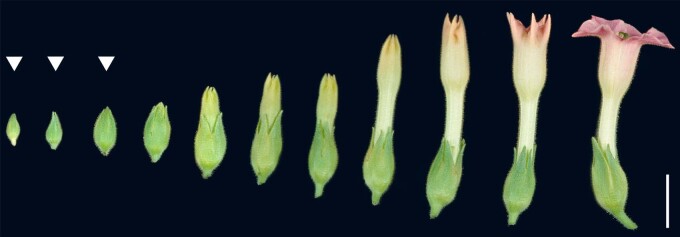
Stress application to developing microspores. Abiotic stress was applied at early stages of development of the male gametophyte. Flower buds ≥10 mm in length were removed from WT^ptGFP^ plants prior to plant transfer to controlled environment chambers for stress application. White arrowheads indicate the developmental window of the flower buds at the time of plant transfer to the stressful condition. Pollen development in these buds (<10 mm long) largely occurs under stress. Scale bar, 10 mm.

Using this experimental system, we applied mild abiotic stress conditions that commonly occur in nature: heat, drought, chilling and light stress. As maternal inheritance is usually established by organelle exclusion and/or organelle degradation during male gametogenesis^33,38^, the stress conditions were specifically applied during pollen development (Fig. 1a). To this end, plants raised under standard growth conditions were transferred to high light stress at 1,000 µE m^−2^ s^−1^, drought stress (by withholding water until visible wilting occurred), heat stress at 35 °C or chilling stress at 10 °C after they had set flower buds (Extended Data Fig. 1). Following completion of pollen development, mature pollen from stressed plants was used to fertilize flowers of unstressed plants. Large-scale crosses were performed for all four stress conditions and for standard conditions in the absence of environmental stress^31^. For each stress condition, between 472,000 and 727,000 seeds were produced (Table 1).

**Table 1:** Crossing experiments and screens for paternal plastid transmission by spectinomycin selection

	Treatment	Abiotic stress applied	Paternal genetic background	Harvest	Seeds screened	No. of paternal transmission events	Paternal plastid transmission rate (%)
Exp. 1	Standard	−	WT^ptGFP^	1	942,992	16	0.0017
				2	238,423	6	0.0025
				3	637,379	10	0.0016
				4	275,794	1	0.0004
				Total	2,094,588	33	0.0016
	High Light	1,000 μE m^−2^ s^−1^	WT^ptGFP^	1	152,719	2	0.0013
				2	146,631	1	0.0007
				3	174,965	0	−
				Total	474,315	3	0.0006
	Heat	35 °C	WT^ptGFP^	1	247,451	0	−
				2	248,615	1	0.0004
				3	231,540	3	0.0013
				Total	727,606	4	0.0005
	Drought	No watering until visible wilting occurred	WT^ptGFP^	1	173,077	2	0.0012
				2	131,329	4	0.0030
				3	167,625	4	0.0024
				Total	472,031	10	0.0021
	Chilling	10 °C	WT^ptGFP^	1	167,169	286	0.1711
				2	147,661	402	0.2722
				3	164,400	463	0.2816
				Total	479,230	1,151	0.2402
Exp. 2	Standard	−	WT^ptGFP^		14,060	0	−
			*dpd1*^ptGFP^		18,060	24	0.1329
	Chilling	10 °C	WT^ptGFP^		9,560	16	0.1674
			*dpd1*^ptGFP^		9,060	198	2.1854
Exp. 3	Chilling	10 °C	WT^ptGFP^		12,000	31	0.2583
			*dpd1*^ptGFP^		12,500	396	3.1680

Seeds were germinated in the presence of spectinomycin (to which only the plastid genome of the father confers resistance), and seedlings with green sectors were identified by visual inspection and observation under the stereomicroscope. To distinguish paternal transmission events from (occasionally appearing) spontaneous spectinomycin-resistant cells^39^, all detected green sectors were examined by UV microscopy to test for expression of the fluorescent reporter protein GFP in plastids (Fig. 1c).

### Chilling stress leads to biparental inheritance of plastids

Screening of progeny obtained by fertilization with pollen from high light-stressed, drought-stressed and heat-stressed plants revealed that all of these crosses showed similar levels of very low paternal leakage as the unstressed control crosses^31^ (Table 1, and Extended Data Tables 1 and 2). By contrast, seedlings obtained from crosses with pollen from chilling-stressed plants displayed strongly elevated levels of biparental inheritance (Fig. 1d and Table 1). Out of 479,230 progeny assayed, 1,151 seedlings inherited paternal plastid genomes, corresponding to a biparental transmission frequency of 0.24% (Table 1). This frequency represents a >150-fold increase in the rate of paternal plastid transmission compared with the unstressed control (Table 1).

Regeneration of green sectors into plants in the presence of spectinomycin resulted in uniformly green plants (referred to as PPI-C lines for paternal plastid inheritance under chilling stress) that were analysed by Southern blotting to directly confirm the presence of the paternal plastid genomes. Restriction fragment length polymorphism (RFLP) analysis revealed that all lines contained the paternal plastid DNA (Fig. 1e). Confocal laser-scanning microscopy confirmed the plastids as the sites of GFP fluorescence (Fig. 1f).

### Paternal plastid transmission in the absence of selection

**Extended Data Fig. 2:**
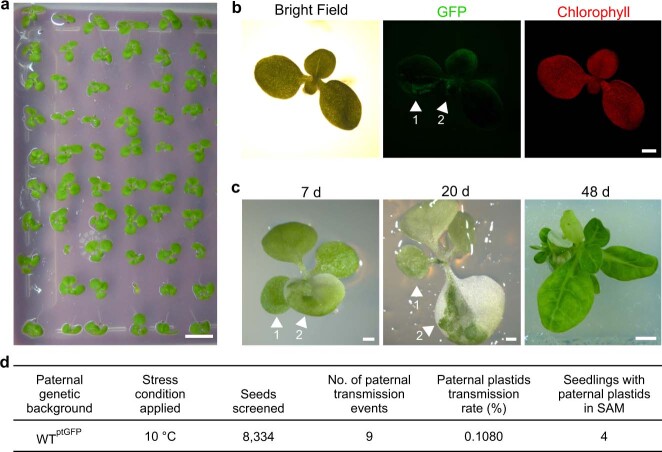
Detection of paternal plastid transmission in the absence of selection. **a**, Seeds harvested from crossing experiments were germinated in medium without antibiotics. Scale bar, 10 mm. **b**, Stereomicroscopic images of a seedling containing paternally inherited plastids grown in antibiotic-free medium. The microscopic bright field picture (left), the GFP fluorescence image (middle) and the chlorophyll fluorescence image (right) are shown. Arrowheads 1 and 2 point to two sectors containing paternal plastid as evidenced by GFP fluorescence. Scale bar, 1 mm. **c**, Visualization of paternal plastids by their antibiotic resistance. The seedling shown in **b** was transferred to spectinomycin-containing medium that causes bleaching of cells that contain only maternal plastids. Photographs were taken 7 days (left; scale bar, 1 mm), 20 days (middle; scale bar, 1 mm) and 48 days (right; scale bar, 10 mm) after transfer. **d**, Frequency of paternal plastid transmission observed in the absence of spectinomycin selection.

The measured frequency of biparental plastid inheritance under chilling stress should be high enough to identify paternal transmission events in the absence of antibiotic selection, by randomly analysing a sufficiently large number of seedlings grown under non-selective conditions. When 8,334 seedlings grown on spectinomycin-free medium were screened for GFP expression, 9 seedlings showed clear GFP fluorescence and 4 had a fluorescent shoot apical meristem (Extended Data Fig. 2). This result confirms the high rate of biparental transmission measured in the spectinomycin selection experiment under chilling stress (Table 1) and, importantly, demonstrates that the selection process does not influence the detection of paternal transmission events.

Subsequent transfer of seedlings displaying fluorescent sectors and/or meristems to spectinomycin-containing medium resulted in bleaching of cells that contain only maternal plastids^40^ and thus directly visualized the presence of inherited paternal plastids (Extended Data Fig. 2c).

### Cytological basis of paternal transmission upon chilling

**Fig. 2:**
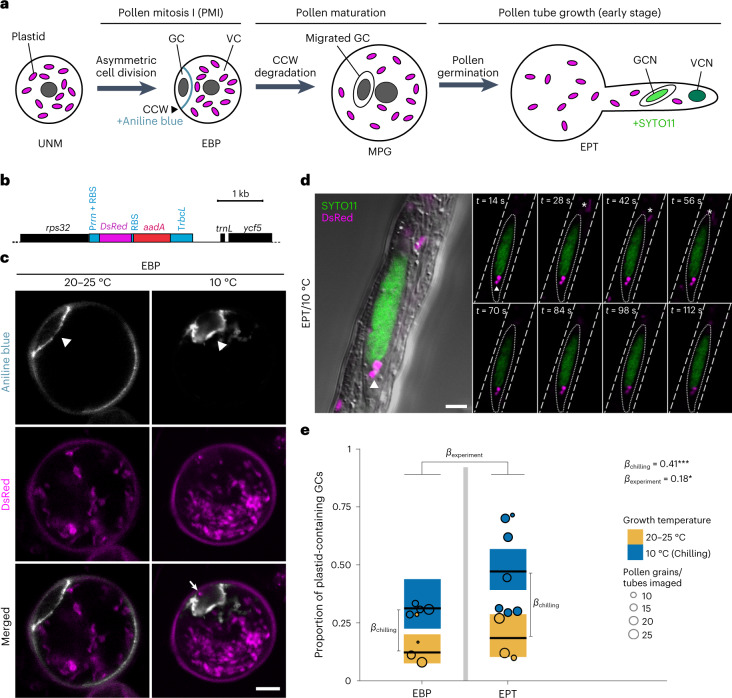
Plastid exclusion in pollen development is compromised under chilling stress. **a**, Schematic diagram of pollen maturation in tobacco. Plastid exclusion is believed to occur in pollen mitosis I by a strongly asymmetric cell division, preventing entry of the vast majority of plastids into the GC in EBP. VC, vegetative cell; GCN, generative cell nucleus; VCN, vegetative cell nucleus. **b**, Map of the transgene-containing region in the ptDsRed plastid genome, harbouring the *DsRed* expression cassette and the spectinomycin resistance gene *aadA*. Expression elements (promoters, terminators and ribosome-binding sites, RBS) are indicated in blue. **c**, Confocal images of EBP in WT^ptDsRed^ plants. The CCW (grey) transiently formed after PMI is visualized by aniline blue staining (arrowheads). The open arrow points to a plastid (magenta) included in the GC in pollen developed at 10 °C. This experiment was repeated four times and representative images are shown. Scale bar, 10 μm. **d**, Time-lapse confocal images of in vitro germinating EPT expressing *DsRed* in plastids. The GCN (green) is visualized by SYTO11 staining. The GC membrane is indicated by a dotted line. Arrowheads and asterisks indicate plastids (magenta) located inside or outside the GC, respectively. This experiment was repeated ten times and representative images are shown. Scale bar, 5 μm. **e**, Proportion of pollen grains with plastids included in the GC. Each circle shows the proportion of plastid-containing GCs in an imaging session/replicate (Extended Data Table 3). Differences between experimental groups are represented by parameter values (*β*) expressed as log_10_ of the fold change, and were estimated using a binomial model (Model 2, *n*_rep.total_ = 18 imaging sessions, 301 grains analysed; Extended Data Tables 1 and 2): *β*_chilling_ represents the effect of the chilling treatment (*P* = 6.01 × 10^−5^); *β*_experiment_ represents differences between the EPT (*P* = 0.0288) and EBP experiments (Extended Data Table 2). Mean estimates and CI95 per experimental group are shown in black horizontal lines and coloured boxes, respectively. Parameters were tested using simultaneous two-tailed Wald *z*-tests. *P < 0.05; ***P < 0.001; *α* = 0.05. Source data

Given that low temperature greatly alters the inheritance mode of plastid genomes, we set out to investigate the effect of chilling stress on the fate of paternal plastids in gametogenesis. During male gametogenesis, a highly asymmetric cell division takes place in pollen mitosis I (PMI; Fig. 2a). Previous studies suggested that all (or the vast majority of) paternal plastids are excluded from the smaller generative cell (GC), resulting in maternal plastid inheritance^33,34^. To test whether chilling stress has a direct impact on this process, we examined the effect of low temperature on paternal plastid segregation during pollen maturation. To this end, two stages in pollen development were investigated by confocal laser-scanning microscopy using a transplastomic line that expresses the DsRed fluorescent reporter inside plastids (Fig. 2b): the early binucleate pollen (EBP, the product of PMI) and the early pollen tube stage (EPT; Fig. 2a). Detection of the DsRed reporter indicated that pollen plastids are capable of active transgene expression (Fig. 2c).

**Extended Data Fig. 3:**
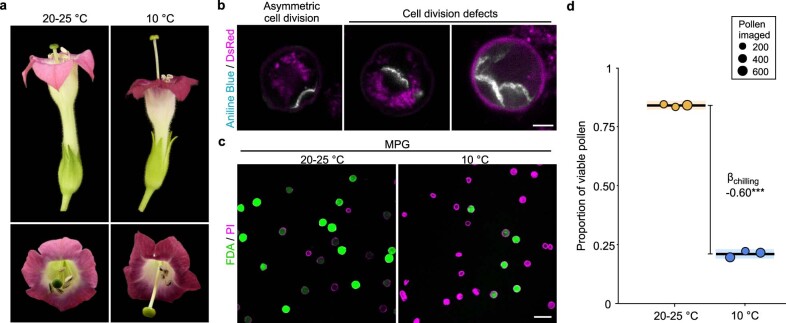
Effect of chilling stress on floral and pollen development in *Nicotiana tabacum*. **a**, Flowers developed under standard greenhouse conditions (left, 20–25 °C) compared to flowers developed under chilling stress (right, 10 °C). **b**, Confocal laser-scanning microscopy images of early binucleate pollen (EBP) harvested from WT^ptDsRed^ plants grown at 10 °C. The callose cell wall separating the generative cell from the vegetative cell was stained with aniline blue to visualize the division pattern. In addition to the normal cell division pattern (left, asymmetric), various defects in cell division (middle and right) are observed in pollen developed at 10 °C. This experiment was repeated four times and representative images are shown. Scale bar, 10 μm. **c**, Confocal microscopy images of mature pollen grains (MPGs) from wild-type tobacco plants grown under standard greenhouse conditions (left, 20–25 °C) or under chilling stress (right, 10 °C). MPGs were stained with fluorescein diacetate (FDA) and propidium iodide (PI) to determine pollen viability. Viable pollen is evidenced by green fluorescence, because FDA is converted to fluorescein by cytoplasmic esterase activity. Dead pollen displays only PI fluorescence inside the grain, due to absence of FDA derived fluorescence and permeability to PI. This experiment was repeated three times and representative images are shown. Scale bar, 100 μm. **d**, Quantification of pollen viability. The proportions of viable pollen per biological replicate (imaging session) were modelled using the binomial distribution (Model 4 constructed with *n*_rep.total_ = 6, 3 sessions per group, ~3100 pollen grains analysed; see Extended Data Tables 1 and 2). Shaded coloured boxes show the 95% confidence interval (CI95) of the mean estimates. *β*_chilling_ represents the effect of the chilling treatment on pollen survival (*P* = 1.64 × 10^−176^), expressed as log_10_ of the fold change (Extended Data Table 2). The parameter estimate was tested by a two-tailed Wald *z*-test. ***, *P* < 0.001; *α* = 0.05.

The newly formed GC in the EBP is bound by a callose cell wall (CCW) that transiently forms after PMI and can be visualized by aniline blue staining (Fig. 2a). Growth at low temperature (10 °C) delayed floral and pollen development, reduced pollen viability and occasionally cause aberrant cell division patterns (Extended Data Fig. 3). Confocal microscopic analysis of EBP at ambient versus low temperature revealed that, at 10 °C, the shape of the GC is irregular and plastids are more frequently included in the cytoplasm of the GC (Fig. 2c,e and Extended Data Table 3; for three-dimensional reconstructions of representative pollen, see Supplementary Videos 1 and 2).

To test whether the plastids persist in later stages of pollen development, GCs were analysed in the pollen tube upon germination (EPT; Fig. 2a) by in vivo time-lapse confocal microscopy. The data revealed that plastids included in the GC during PMI under chilling conditions were still present upon GC migration into the pollen tube (Fig. 2a,d and Supplementary Video 3), thus probably contributing to the substantially increased paternal plastid transmission at low temperature (Fig. 2e and Extended Data Tables 1–3).

It is important to note that the observed increase in the entry of paternal plastids into the GC is insufficient to fully explain the >150-fold increase in chilling-induced paternal plastid transmission (cf. Figs. 1d and 2e). Thus, in addition to cell division and organelle distribution, chilling probably affects other cellular processes that are relevant to organelle inheritance. As low temperature also reduces the activities of all enzymes in the cell, we therefore considered candidate enzymes that could be involved in plastid inheritance and whose reduced activity at 10 °C can potentially explain the increase in paternal transmission that remains unaccounted for by organelle distribution in PMI alone.

### Control of plastid inheritance by the exonuclease DPD1

**Fig. 3:**
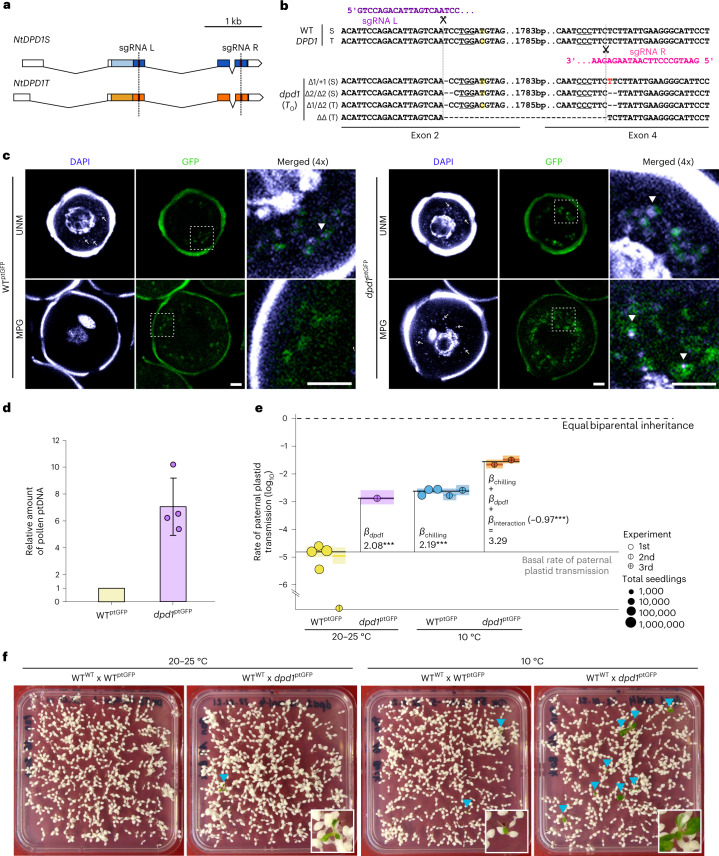
NtDPD1 controls plastid DNA degradation and inheritance. **a**, Transcript maps of *DPD1* homeologues. *N. tabacum* harbours two *DPD1* genes: *NtDPD1S* and *NtDPD1T* (coding regions in blue and orange, respectively). black lines, introns; boxes, exons; dark colour, conserved exonuclease domain; dashed lines, target sites of sgRNAs L and R. **b**, Genomic sequences of *DPD1* wild-type loci (top) and of a *dpd1* mutant generated using CRISPR/Cas9 (bottom). PAM sequences are underlined. Yellow shades denote polymorphic bases between homeologues. ΔΔ symbolizes a large deletion. **c**, Confocal images of WT^ptGFP^ (left) and *dpd1*^ptGFP^ (right) pollen. UNM and MPG were stained with DAPI to visualize nuclear and plastid DNA. Arrows indicate organellar DNA. In the merged channels (right: ×4 enlargement of the dotted boxed area), arrowheads indicate overlapping DAPI and ptGFP fluorescence signals. The images are representative of three experiments. Scale bar, 10 μm. **d**, Relative quantification of plastid DNA in enriched pollen fractions of WT^ptGFP^ and *dpd1*^ptGFP^. Samples were measured in triplicate by real-time PCR; mean plastid DNA amounts (*aadA* amplicon) were normalized to mean nuclear DNA amounts (*18S rDNA* amplicon). Values for *dpd1*^ptGFP^ (circles) are relative to the corresponding WT^ptGFP^ samples from four replicates using different plants. Error bars, ±s.e.m. **e**, Rates of paternal plastid transmission across experiments. Circles represent proportion of seedlings per harvest that carry green sectors. Effects of genotype and chilling treatment across the three independent experiments (Table 1) were analysed in Model 3 (*n*_rep.total_ = 13 harvests, ~2.65 million seedlings; Extended Data Tables 1 and 2). Black horizontal bars show mean rates per genotype/treatment combination. Rates per experimental group were estimated (coloured horizontal lines) with CI95s (coloured boxes). Dashed lines depict the basal plastid paternal transmission (grey) and the theoretical maximum (black). Effect estimates were tested by simultaneous two-tailed Wald *z*-tests: *dpd1* genotype (*P* = 6.22 × 10^−35^), chilling treatment (*P* = 2.20 × 10^−126^) and the interaction between both factors (*P* = 3.17 × 10^−10^) were significant. ***P < 0.001, *α* = 0.05. **f**, Visualization of paternal plastid transmission by spectinomycin selection (Experiments 2 and 3; Table 1). Blue arrowheads indicate green sectors (paternal plastids). Insets show magnified examples.

**Extended Data Fig. 4:**
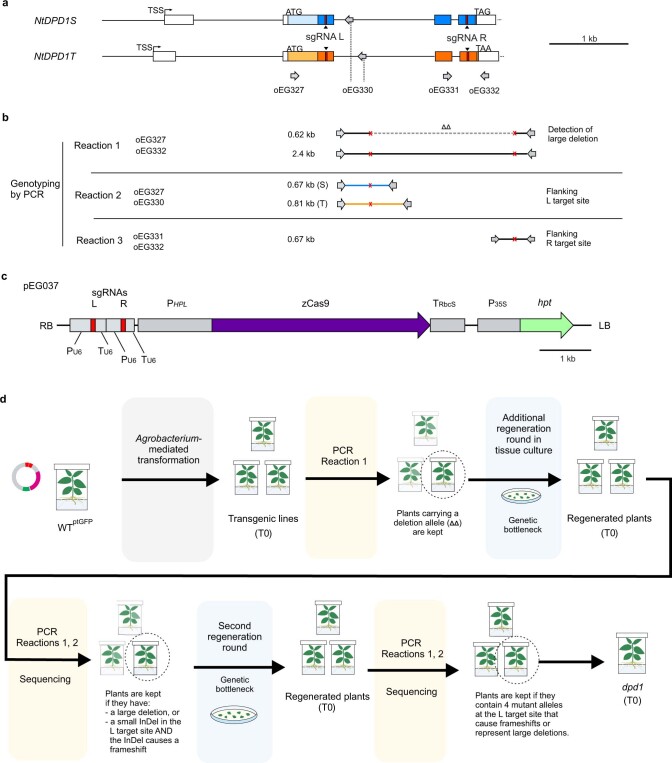
Generation of *dpd1* mutants in the allotetraploid species *Nicotiana tabacum*. **a**, Primer (oEG) binding sites in the *NtDPD1* genomic sequence. Boxes represent exons. TSS is the transcription start site according to transcriptome data. Blue and orange areas indicate the protein-coding sequences in the two diploid subgenomes (*S: Nicotiana sylvestris; T: Nicotiana tomentosiformis*). The conserved exonuclease domain is represented by the darker color. The sequences targeted by CRISPR/Cas9-based genome editing (sgRNA-binding sites; sgRNA L: ‘left’ sgRNA; sgRNA R: ‘right’ sgRNA) are indicated by red vertical bars and black arrowheads. Start and stop codons are also shown. **b**, PCR reactions used for genotyping of the *DPD1* loci. Red marks represent the mutations produced by Cas9 cleavage at the L and R target sites. Large deletions (ΔΔ) are the result of double cleavage and loss of the fragment between the L and R sites. **c**, Structure of the T-DNA in transformation vector pEG037 used for mutagenesis of *NtDPD1*. RB and LB are the T-DNA borders. P_U6_ and T_U6_ are promoter and terminator, respectively, of the U6 snRNA gene from *Arabidopsis thaliana*. The sgRNAs R and L are shown as red boxes. P_*HPL*_ is the promoter from the *HPL* gene of *A. thaliana* (AT4G15440). zCas9 is a *cas9* gene version that was codon-optimized for *Zea mays*. T_RbcS_, Rubisco small subunit terminator; P_35S_, double CaMV 35S promoter; *hpt*, hygromycin phosphotransferase gene conferring resistance to hygromycin *in planta*. **d**, Schematic overview of the generation of *dpd1* mutants. After *Agrobacterium*-mediated transformation with vector pEG037, PCR-based genotyping and additional regeneration cycles in tissue culture were conducted. Somatic expression of Cas9 from the *HPL* promoter potentially causes different mutations in different parts of the plants. Plants with putative knock-out alleles of *DPD1* were identified by PCR genotyping and DNA sequencing, and the additional regeneration rounds were performed to reduce the mosaicism in the mutant lines by obtaining clonal regenerants from single cells. The procedure led to the isolation of plant lines in which all cells contain the same set of mutated *DPD1* alleles (*dpd1* mutant).

During male gametogenesis, plastid DNA is actively degraded, and the exonuclease *At*DPD1 plays a key role in this process in *Arabidopsis thaliana*^41^. Thus far, *At*DPD1 has not been shown to affect maternal inheritance^41^ and instead, has been suggested to facilitate phosphate mobilization from plastid DNA under phosphorus starvation conditions^42^. Having seen that low temperature promotes inclusion of plastids in the GC, we hypothesized that escape from genome degradation can also enhance the rate of paternal plastid genome transmission. To test this idea, we set out to produce a *dpd1* knockout mutant in tobacco by genome editing. Tobacco (*N. tabacum*) is an allotetraploid species comprising the genomes of two diploid species, *N. sylvestris* and *N. tomentosiformis*, thus necessitating generation of a tetra-allelic knockout (Fig. 3a,b and Extended Data Fig. 4). Targeting of the *dpd1* alleles in both subgenomes by CRISPR/Cas9 editing with appropriately designed sgRNAs (single guide RNAs) allowed isolation of a *dpd1* null mutant (see Methods, Fig. 3a,b and Extended Data Fig. 4).

**Extended Data Fig. 5:**
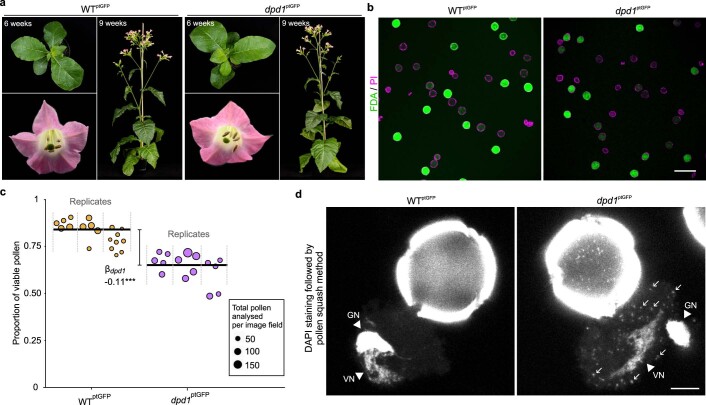
Phenotypic analysis of the tobacco *dpd1* mutant. **a**,WT^ptGFP^ and *dpd1*^ptGFP^ plants were grown in the greenhouse. Pictures were taken from 6-week-old plants, 9-week-old plants and fully opened flowers. **b**, Confocal microscopy images of pollen grains from WT^ptGFP^ and *dpd1*^ptGFP^ plants grown under standard greenhouse conditions. Pollen grains were stained with fluorescein diacetate (FDA) and propidium iodide (PI) to determine pollen viability. This experiment was repeated three times and representative images are shown. Scale bar, 100 μm. **c**, Quantification of pollen viability. The proportions of viable pollen per biological replicate (imaging session) were modelled using the binomial distribution (Model 5; constructed with *n*_rep.total_ = 6; 2,228 pollen grains analysed; see Extended Data Tables 1 and 2). Each replicate consisted of a single harvest of pollen from 2–3 flowers of a single plant, which were stained and imaged in the same session (delimited by dashed lines). During data analysis, it was found that there was significant variation between replicates (represented by replicate parameters with *P* < 0.05 in Extended Data Table 2). To better represent this variation, the graph shows the proportions of viable pollen in the 5–9 image fields (coloured circles) photographed in each replicate. The mean estimates per genotype are indicated with a black horizontal bar. *β*_*dpd1*_ represents the effect of the *dpd1* genotype on pollen survival (*P* = 3.00 × 10^−9^) compared to the wild type at normal temperature conditions (20–25 °C), expressed as log_10_ of the fold change (Extended Data Table 2). The parameter estimate was tested by a two-tailed Wald *z*-test. ***, *P* < 0.001; *α* = 0.05. **d**, Confocal images of WT^ptGFP^ and *dpd1*^ptGFP^ mature pollen stained with DAPI. Images were taken after applying the pollen squash method to release the mature pollen from the anthers. Z-stack overlaid images with maximized projection are displayed (total width of z-slices of WT^ptGFP^ and *dpd1*^ptGFP^ is 2.15 μm and 1.78 μm, respectively). Arrowheads indicate the vegetative nucleus (VN) and the generative nucleus (GN). Arrows indicate organellar DNA released from squashed mature *dpd1*^ptGFP^ pollen. This experiment was repeated two times and representative images are shown. Scale bar, 10 μm.

Consistent with previous findings reported in *A. thaliana*, the tobacco *dpd1* mutant does not display a noticeable vegetative growth phenotype. Plant growth, reproductive transition and floral development in the *dpd1* mutant were comparable to wild-type plants (Extended Data Fig. 5a). However, a reduction in pollen viability was observed in the *dpd1* mutant (Extended Data Fig. 5b,c). Confocal laser-scanning microscopy and quantitative PCR analysis revealed retention of plastid DNA in mature pollen grains of the *dpd1* mutant (Fig. 3c,d and Extended Data Fig. 5d). Large-scale inheritance assays showed that the *dpd1* mutant displayed greatly elevated levels of paternal plastid transmission into the progeny (Fig. 3e,f and Table 1), thus identifying the rate of plastid genome degradation as a genetic factor determining the mode of plastid inheritance.

### Synergistic gene–environment control of plastid transmission

Next, we wanted to examine whether the combined action of genes and environment confers even higher levels of biparental plastid inheritance. To this end, we comparatively assessed the effects of chilling stress on the transmission frequency of paternal plastids in the wild-type and the *dpd1* genetic backgrounds. Indeed, pollen development at low temperature in the *dpd1* mutant resulted in an approximately tenfold increase in paternal plastid transmission compared with the effect of the *dpd1* genotype alone or that of the environment alone (Table 1 and Fig. 3e,f). Paternal plastid transmission reached frequencies between 2.2% and 3.2% (in two independent experiments; Fig. 3e and Table 1). These data show that genes and environment act synergistically in the control of plastid inheritance, and their combined action can result in substantial levels of biparental inheritance (Fig. 3e). It is also noteworthy that the environmental factor low temperature and the genetic factor *dpd1* are not entirely independent in their effects on plastid inheritance, as revealed by the negative interaction between them (Fig. 3e).

### Paternally transmitted plastids readily enter the germline

**Fig. 4:**
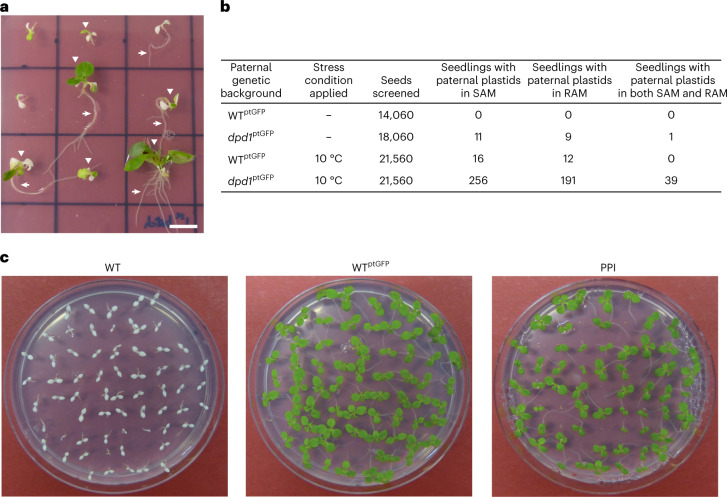
Transmission of paternal plastids into apical meristems and their inheritance in the next generation. **a**, Images of seedlings with inherited paternal plastids present in the shoot apical meristem (SAM, white arrowheads) or root apical meristem (RAM, white arrows), as evidenced by growth in the presence of spectinomycin. Scale bar, 10 mm. **b**, Quantification of paternal plastid transmission into SAMs and RAMs. Data were obtained from crossing experiments 2 and 3. **c**, Seed germination assays to confirm the inheritance of the paternally transmitted plastid into the next generation. Seeds from a WT plant, a transplastomic WT^ptGFP^ plant and a line with PPI were germinated on medium with 500 μg ml^−1^ spectinomycin.

Biparental inheritance events in which the inherited paternal plastids are present in the shoot apical meristem (Extended Data Fig. 2b,c and Fig. 4a,b) are particularly relevant in that from there, they enter the germline (upon transition of the apical meristem into a floral meristem) and become heritable. Therefore, large-scale screens were conducted to quantitatively assess the entry of paternal plastids into shoot and root apical meristems in the wild-type and the *dpd1* genetic backgrounds under ambient temperature or chilling stress. Presence of paternal plastids was evidenced by GFP fluorescence in the meristem (Extended Data Fig. 2b,c) and continued plant growth in the presence of spectinomycin (Fig. 4a). The presence of paternal plastids in the shoot apical meristem was observed at high frequency, in almost half of the biparental transmission events in *dpd1* under chilling stress (cf. Table 1 and Fig. 4b).

To determine whether the inherited paternal plastid genomes can be stably maintained across generations, plants with paternal plastids present in the shoot apical meristem were grown in the greenhouse and self-pollinated for seed production. Uniform resistance of the progeny to spectinomycin (Fig. 4c) demonstrated homoplasmy of the (paternally acquired) plastid genome, which is maternally inherited into the next generation in the absence of stress. Together, these data show that paternally inherited plastids readily enter the germline and are passed on to the next generation, suggesting that the discovered phenomenon has evolutionary significance for adaptation^43^ and, potentially, speciation^44^.

## Discussion

**Fig. 5:**
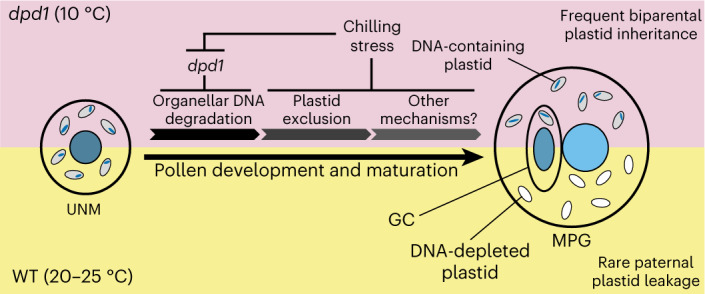
Mechanistic model of uniparental versus biparental plastid inheritance. The combined effects of *dpd1* and low temperature promote paternal plastid transmission, resulting in a shift from maternal to biparental inheritance of plastids and their genomes.

In the course of this work, we have discovered two distinct factors that jointly determine plastid inheritance in plants. While the environmental factor temperature affects plastid distribution during the asymmetric cell division occurring in PMI, the genetic factor *DPD1* affects organellar genome degradation during male gametogenesis (Fig. 5).

Surprisingly, the possibility that plastid inheritance is influenced by the environment has not been considered previously. Here we discovered that maternal inheritance breaks down when male gametogenesis occurs at low temperature. We identified the underlying mechanism by showing that chilling promotes inclusion of paternal plastids in the GC. Chilling stress has been shown to result in destabilization of the cytoskeleton during gametogenesis^45^. The occurrence of altered cell division patterns in PMI upon plant exposure to low temperatures (Extended Data Fig. 3b) suggests that compromised cytoskeletal function is responsible for incomplete plastid exclusion from the GC.

The second elimination mechanism uncovered by our work acts at the level of organellar genome stability. The exonuclease DPD1 degrades organellar genomes in maturing pollen, thus providing a fail-safe mechanism that genetically inactivates those plastids that escaped the organelle exclusion mechanism operating in PMI. Consequently, when both mechanisms (plastid exclusion and genome degradation) are compromised, substantial levels of biparental inheritance ensue (Figs. 3e and 5). Together, our findings suggest that plastid inheritance is a complex trait that is affected by both genetic and environmental factors.

Although most organisms inherit their plastids and/or mitochondria maternally, biparental inheritance has evolved multiple times independently in both animals and plants^46^. It will be interesting to investigate species that exhibit biparental inheritance of plastids and/or mitochondria, and determine the expression patterns of genes for cytoskeletal proteins (especially those that have been implicated in organelle movement^47–49^) and the activity of organelle-targeted nucleases during male gametogenesis. DPD1 homologues are present in both angiosperms and gymnosperms^42^, and the cytoskeletal components are also generally highly conserved. These candidate genes and their expression patterns could also explain the variation in the rates of biparental plastid inheritance in natural populations^20^, and the switches in inheritance modes seen over evolutionary timescales^2^.

Given the high conservation of the male gametogenesis programme in seed plants, we speculate that the mechanisms identified in our work are likely to be relevant to all seed plant species. However, it is important to note that the mechanisms reported here are primarily operating during PMI. It seems possible that maternal factors and/or post-fertilization mechanisms also contribute to the control of cytoplasmic inheritance in plants.

Our work also provides simple methods to alter organellar inheritance patterns. In the light of our findings, shifts to biparental inheritance can be achieved either temporarily (by applying chilling stress) or permanently (by genetic interventions, for example, *DPD1* gene editing). Changing the mode of inheritance of organelles has important applications in plant breeding. In most crops, both plastids and mitochondria are maternally inherited, making it notoriously difficult to separate the influence of the plastid from that of the mitochondrial genome on important phenotypic traits such as growth, yield and stress tolerance^23,43,50,51^. Induction of biparental transmission will allow separation of plastid from mitochondrial effects by taking advantage of the random segregation and sorting of the two organelle types in the progeny.

Finally, our finding that mild chilling stress triggers substantial rates of biparental plastid inheritance has far-reaching consequences for our understanding of organellar genome evolution. Low temperatures (10 °C) are ubiquitous in nature, hence our findings provide a possibility for organelles to participate frequently in sexual reproduction. Assuming that our findings reported here extend to mitochondrial inheritance, the paternally transmitted mitochondria would be able to fuse with their maternal counterparts, leading to genome recombination^52,53^. This could efficiently counteract Muller’s ratchet by generating new variation in mitochondrial genomes and facilitating the combination of favourable mutations.

In contrast to plant mitochondria, fusion and genome recombination in plastids appear to be rare^17–19^. However, temperature-induced biparental inheritance also provides a mechanism for plastids to spread through pollen between species and populations, a phenomenon known as chloroplast capture^22,54^. Interestingly, accumulating evidence suggests that the plastid genome plays roles in tolerance to low temperatures^55,56^, thus potentially providing a direct link between chilling-induced biparental plastid inheritance, selection and adaptation.

In summary, our data reported here demonstrate that plastid inheritance is controlled at two distinct steps in male gametogenesis and determined by synergistic gene–environment interaction. Our finding that uniparental inheritance of plastids breaks down under conditions of mild environmental stress indicates that organellar genomes experience episodes of biparental inheritance, and casts considerable doubt on the long-held tenet that organelles are asexual genetic systems.

## Methods

### Plant material and growth conditions

Tobacco plants (*Nicotiana tabacum* cv. Petit Havana) were grown in standard greenhouse conditions at ~300 μE m^−2^ s^−1^ light intensity under a 16 h light/8 h dark regime (day temperature: ~25 °C, night temperature: ~20 °C). Transplastomic lines (with a wild-type nuclear background; WT^ptGFP^) used as pollen donor harbour a *gfp* expression cassette and the selectable marker *aadA* encoding an enzyme conferring spectinomycin resistance^31^. Transplastomic lines (WT^ptDsRed^) used for confocal microscopic studies harbour a DsRed expression cassette (driven by the ribosomal RNA operon promoter and a chimeric 5′ untranslated region from the chloroplast *psbA* and *clpP* genes) and the spectinomycin resistance marker *aadA*^57^. Seed germination and plant cultivation in vitro were performed on agar-solidified synthetic medium containing 3% (w/v) sucrose^58^. Abiotic stress treatments were performed in controlled-environment chambers.

### Crossing experiments and screening for paternal plastid transmission

Homoplasmic transplastomic plants were raised under standard greenhouse conditions and transferred to the designated abiotic stress condition in controlled-environment chambers upon onset of flower development (Fig. 1a). To ensure that male gametophyte development occurred under stress, flower buds ≥10 mm in length were removed upon transfer (Extended Data Fig. 1). High light stress was applied by shifting plants to 1,000 μE m^−2^ s^−1^ light intensity. Drought stress was applied by withholding water until visible loss of turgor and wilting occurred. Heat stress was applied by shifting plants to 35 °C, and chilling stress was performed by shifting plants to 10 °C. Upon pollen maturation, large-scale crosses were conducted by manual pollination^31^, using pollen from transplastomic plants (WT^ptGFP^ or *T*_1_ *dpd1*^ptGFP^) grown in standard greenhouse conditions or under abiotic stress. Greenhouse-grown plants with wild-type plastids were used as maternal recipients: male-sterile Nt-*nms* plants in Experiment 1^31^, and emasculated wild-type plants in Experiments 2 and 3.

The resulting progeny was assayed for paternal plastid transmission by germinating seeds on synthetic medium in the presence of spectinomycin (500 µg ml^−1^), at a density of approximately 500 seedlings per 180-mm-diameter Petri dish or 120 mm square plate. Seedling phenotypes were analysed by visual screening under a stereomicroscope (Zeiss), followed by inspection of detected green (antibiotic-resistant) sectors by UV and confocal microscopy to test for GFP fluorescence. Paternal plastid inheritance (PPI) lines were transferred to soil, grown to maturity in the greenhouse and selfed for seed production. Seeds were assayed for stable paternal plastid inheritance by germination on agar-solidified synthetic medium with spectinomycin (500 µg ml^−1^).

### Tissue culture and plant regeneration

To regenerate plants that contain paternal plastids, green (spectinomycin-resistant) sectors were excised from cotyledons or primary leaves and regenerated on agar-solidified plant regeneration medium^58^ containing 3% (w/v) sucrose, 0.1 mg ml^−1^ 1-naphtaleneacetic acid (NAA), 1.0 mg ml^−1^ 6-benzylaminopurine (BAP) and 500 µg ml^−1^ spectinomycin. To eliminate spontaneous spectinomycin-resistant mutants, tissue samples were exposed to double selection on medium containing spectinomycin and streptomycin (500 µg ml^−1^ each). Spontaneous spectinomycin-resistant cells bleach out on this medium, whereas cells with *aadA*-expressing transgenic plastids remain green and continue to grow^59,60^. To obtain seeds, regenerated homoplasmic lines with paternal plastids were rooted and propagated on synthetic medium in the presence of spectinomycin (500 µg ml^−1^).

### Light microscopy, UV microscopy and confocal laser-scanning microscopy

Green sectors potentially harbouring paternal plastids were identified in germinating seedlings by visual inspection and/or light microscopy using a stereomicroscope (Stemmi 2000-C; Zeiss). GFP fluorescence was detected with an MZ FLIII fluorescence stereomicroscope (Leica) using filters GFP2 (excitation filter: BP 480/40 nm, barrier filter: LP 510 nm) and GFP3 (excitation filter: BP 470/40 nm, barrier filter: BP 525/50 nm). Subcellular localization of GFP fluorescence was determined by UV microscopy with an Axioskop 2 (Zeiss; excitation filter: BP 450/90 nm, barrier filter: BP 515/65 nm), or by confocal laser-scanning microscopy (TCS SP8; Leica) using an argon laser for excitation (at 488 nm), a 500–510 nm filter for detection of GFP fluorescence and a 610–700 nm filter for detection of chlorophyll fluorescence. Subcellular localization of DsRed fluorescence was determined by confocal laser-scanning microscopy (TCS SP8; Leica) using a diode-pumped solid-state laser for excitation (at 561 nm), and a 575–605 nm filter for detection of DsRed fluorescence.

### Aniline blue staining

To visualize the transient CCW, EBP grains were extracted from flower buds with size of 15–18 mm (plants grown at 20–25 °C) or 32–35 mm (plants grown at 10 °C). The CCW was stained by decolorized aniline blue solution (0.1% (w/v) aniline blue (Sigma) in 0.1 M K_3_PO_4_ (pH 11.0)) at room temperature for 10 min. Stained samples were imaged by confocal laser-scanning microscopy (TCS SP8; Leica) using an argon laser for excitation at 405 nm, and a 494–544 nm filter for detection of fluorescence emission. *Z*-stack imaging was performed with a Leica TCS SP8 using the Galvo flow system and optimized settings with a step size of ~0.34 µm.

### SYTO11 staining of in vitro germinating pollen tubes

To visualize the nuclear DNA of the generative cell within pollen tubes, two to three anthers were detached from flowers at anthesis and collected in a 2 ml Eppendorf tube. Mature pollen grains were released from the stamen by vortexing. Subsequently, the stamens were discarded and 100 µl pollen germination buffer (1.6 mM boric acid, 3.0 mM calcium nitrate, 1.0 mM potassium nitrate, 0.8 mM magnesium sulfate, 10% sucrose, pH 7.4) supplemented with 1 µM SYTO11 stain (Thermo Fisher) were added to the pollen. Incubation was done in the dark at room temperature for 120–180 min. Stained pollen tubes were imaged by confocal laser-scanning microscopy (TCS SP8; Leica) using a 488 nm argon laser for excitation and a 500–550 nm filter for detection of the SYTO11 signal.

### Pollen viability assay

To determine the effect of temperature on pollen viability, anthers were detached from flowers at anthesis and collected in a 5 ml Eppendorf tube. Pollen grains were harvested by vortexing with pollen harvesting buffer (100 mM Na_3_PO_4_ (pH 7.0) and 1 mM Na_2_EDTA (pH 8.0)), followed by centrifugation at 1,000 × g for 5 min. The pollen pellet was then resuspended and stained in pollen viability solution (100 mM Na_3_PO_4_ (pH 7.0), 1 mM Na_2_EDTA (pH 8.0), 10 μM propidium iodide (Abcam) and 20 μg ml^−1^ fluorescein diacetate Sigma) at room temperature for 30 min. Stained samples were imaged by confocal laser-scanning microscopy (TCS SP8; Leica). Fluorescein diacetate was excited with a 488 nm argon laser and the emission signal was collected at 500–550 nm. Propidium iodide was excited with a 561 nm argon laser and its emission signal was collected at 600–650 nm.

### DAPI staining

To visualize nuclear and organellar DNA, uninucleate microspores (UNM) and mature pollen grains (MPG) were extracted from flower buds with sizes of approximately 10 mm (for UNM) or from flowers at anthesis (for MPG). DNA was stained with 4,6-diamidino-2-phenylindole (DAPI) staining solution (100 mM Na_3_PO_4_ (pH 7.0), 0.1% (v/v) Triton X-100, 1 mM Na_2_EDTA (pH 8.0) and 1 μg ml^−1^ DAPI) at room temperature for 30 min. Stained samples were imaged by confocal laser-scanning microscopy (TCS SP8; Leica) using a 405 nm laser diode for excitation and a 430–495 nm filter for detection of fluorescence emission. For the pollen squash method, stained pollen grains were placed on a glass slide and gently squashed by tapping on the cover slip^41^. Squashed pollen were imaged by confocal microscopy (Leica TCS SP8), with *z*-stack imaging using the Galvo flow system and optimized settings with a step size of ~0.30 µm.

### *NtDPD1* gene identification and sgRNA design for genome editing

The protein sequence of DPD1 from *Arabidopsis thaliana* (AT5G26940) was used as query in a search against the genomic scaffolds in the available draft genome of *N. tabacum*^61^. Two homologues were identified and named *NtDPD1S* and *NtDPD1T* (scaffolds Nitab4.5_0002715 and Nitab4.5_0014337, respectively). The transcript structures of both loci were deduced from the sequences present in the associated transcriptome (Nitab4.5_0002715g0070.1 and Nitab4.5_0014337g0020.1). Both *NtDPD1* genomic sequences were divided into 2 kb fragments and used as input for sgRNA generation using CRISPOR^62^. A pair of sgRNAs was chosen for *NtDPD1* gene editing (sgRNA L1: 5′-GTCCAGACATTAGTCAATCC...-3′; sgRNA R1: 5′- GATGCCCTTCAATAAGAGAA...-3′, with nucleotides 2–20 corresponding to the target sequence). The targeted sequences are fully conserved in both *NtDPD1* loci.

### Construction of a plant transformation vector for genome editing of *NtDPD1*

Plasmid pEG001 was assembled for constitutive expression of *cas9* in plant cells. pEG001 is a derivative of plasmid pJF1031 described previously^63^. The backbone of pJF1031 was excised by digestion with the restriction enzymes SpeI and XbaI and purification of the ~14.5 kb fragment obtained, resulting in the removal of the promoter driving *cas9*. The ~1.5 kb promoter of the *HPL* gene of *A. thaliana* (hydroxyperoxide lyase, AT4G15440) was amplified from the pORE E2 plasmid^64^ using primers oEG126 and oEG127 (Extended Data Table 4), and integrated into the pJF1031 backbone through a Gibson assembly reaction^65^ (New England Biolabs), resulting in plasmid pEG001. For gene editing at the *NtDPD1* loci, vector pEG037 was constructed. Primers oEG315 and oEG320 (Extended Data Table 4) were used to amplify a fragment of plasmid pJF1046^63^ containing part of an sgRNA scaffold, the U6 promoter and the U6 terminator. The primers introduce the targeting sequences of sgRNA L or R, respectively, as well as BsaI sites at both ends of the fragment. The PCR product was cloned into pEG001 through a simultaneous BsaI restriction and ligation reaction^66^. The resulting binary vector pEG037 for mutagenesis of *NtDPD1* contains a hygromycin resistance marker for selection in planta and a kanamycin marker for selection in bacteria. Sequences of all oligonucleotides used in this study are provided in Extended Data Table 4.

### Generation of a quadruple *dpd1* knockout line

Transplastomic plants with a wild-type nuclear background (WT^ptGFP^) were supertransformed with *Agrobacterium tumefaciens* strain GV2260 harbouring vector pEG037 using the leaf disc infiltration method. Selected hygromycin-resistant plant lines were maintained in medium containing hygromycin (15 mg l^−1^) and cefotaxime (250 mg l^−1^) for continued selection of transgenic plant cells and elimination of residual *Agrobacterium* cells, respectively. Extended Data Fig. 3 illustrates the PCR-based genotyping (Reactions 1–3) of transgenic lines and the screening procedure that led to the isolation of the double *dpd1* mutant (tetra-allelic). Due to the allotetraploidy of the *N. tabacum* genome, the screening process was tailored to identify plants with four knockout *DPD1* homeoalleles in the diploid state (that is, two S and two T alleles; Fig. 4a). PCR and sequencing were employed to identify plants with somatic Cas9 activity and desirable knockout mutations in *dpd1* (Extended Data Fig. 4). Additional regeneration steps in tissue culture helped with purification of desired mutation events and reduction of the allele complexity arising from constitutive Cas9 expression.

Polymorphisms between the S and T loci allowed classification of the amplified sequences as S or T homeoalleles after sequencing (Fig. 3a,b and Extended Data Fig. 4). Reaction 1 (Extended Data Fig. 4b) generates a short product only when a large deletion was generated between targeting sites L and R. Presence of this product implies that the plant line possesses an active Cas9, and at least one mutant allele can confidently be identified by this PCR. The primers used in Reaction 2 (Extended Data Fig. 4b) flank the site targeted by sgRNA L. This reaction was performed to identify the other three mutant alleles by direct Sanger sequencing of amplified PCR products from ~320 *T*_0_ samples analysed. Occurrence of small InDels often caused Reaction 2 to produce electropherograms showing several overlapping peak profiles. Whenever profiles of up to three overlapping sequences were obtained, deconvolution of sequence strings was attempted by visual inspection. At the L site, Cas9 frequently caused small InDels (from −4 to +1 bp) and produced recognizable shifted peak patterns compared to the known S and T wild-type sequences, which could be deconvoluted even when three sequences were overlapping. After three rounds of PCR screening and two rounds of regeneration in tissue culture, a double *dpd1* mutant plant line was isolated (for simplicity, referred to as *dpd1*). Sanger sequencing confirmed that *dpd1* possesses four distinct mutant alleles at the L site (Fig. 3a,b and Extended Data Fig. 4). One of the sequences was a large deletion, and the three other sequences were frameshift mutations.

The *T*_0_ *dpd1* plant was transferred to the greenhouse where *T*_1_ seeds were obtained by selfing. Presence of the mutations at the L site was confirmed by genotyping *T*_1_ seedlings (performing Reactions 1 and 2; Extended Data Fig. 4b). Allele segregation in the *T*_1_ generation facilitated the individual sequencing of the four alleles after PCR and gel purification, as Reaction 2 yields products of different size for the S and T loci. Mutations at the sgRNA R site were analysed in *T*_1_ seedlings by Reaction 3 (Extended Data Fig. 4b), applying visual deconvolution when necessary. The linkage between mutations in the L and R target sites was established by observing co-segregation of genotyped alleles in the *T*_1_ generation (23 seedlings analysed). No other alleles were identified in the final *dpd1* line, suggesting that the four mutant alleles detected in the *T*_0_ generation had become fixed.

### Isolation of nucleic acids and PCR

For DNA gel blot analysis, total plant DNA was isolated using a cetyltrimethylammoniumbromide (CTAB)-based protocol^67^. Extracted DNA samples were digested with the restriction enzymes XhoI and EcoRV, separated by gel electrophoresis in 0.8% agarose gels and blotted onto Hybond N nylon membranes (GE Healthcare) using standard protocols. For hybridization, α[^32^P]dCTP-labelled probes were generated by random priming (Multiprime DNA labelling kit, GE Healthcare). A PCR product covering part of the 16S rRNA gene (amplified by primers P16Srrn-F and P16Srrn-R, and purified by agarose gel electrophoresis) was used as probe for RFLP analysis. Hybridizations were carried out at 65–68 °C in rapid hybridization buffer (GE Healthcare) following the manufacturer’s instructions.

For genotyping reactions, genomic DNA was extracted from leaf tissue with the Extract-N-Amp kit (Sigma-Aldrich). One µl was used as template for PCR. For cloning and for the initial PCR-based screening of *dpd1* mutations, Phusion DNA polymerase (Thermo Fisher) was used. In all other PCR reactions, DreamTaq DNA polymerase (Thermo Fisher) was used. PCR products were column-purified before sequencing (NucleoSpin Gel and PCR Clean-up, Macherey-Nagel).

### Quantification of plastid DNA in pollen

Total DNA (comprising both nuclear and organellar DNA) of enriched pollen fractions was prepared using a published protocol^41^. Tobacco pollen grains were collected by rubbing dehiscent anthers from three opened flowers (WT^ptGFP^ and *dpd1*^ptGFP^ plants, respectively) onto the wall of a 1.5 ml Eppendorf tube, followed by addition of 100 µl of distilled water for resuspension. Due to the procedure used for pollen harvest, some level of contamination by other anther cell types cannot be avoided, hence the samples should be considered as enriched pollen preparations (rather than pure pollen fractions). The enriched pollen suspension was incubated at 95 °C for 5 min, centrifuged at 16,000 x *g* for 5 min and then subjected to real-time PCR to determine the relative amounts of nuclear and plastid DNA. *18S rDNA* (nuclear gene) and *aadA* (transgene present in the plastid genome of WT^ptGFP^ and *dpd1*^ptGFP^) were used as proxies for nuclear and plastid DNA abundance, respectively. Primers oCK68 and oCK69 were used for *18S rDNA* amplification, and primers oKPC579 and oKPC580 for *aadA* amplification (Extended Data Table 4). Quantitative real-time PCR was conducted using the LightCycler 480 Real-Time PCR system and LightCycler SYBR Green reaction mixtures (Roche). The relative amount of plastid DNA in the enriched pollen fractions was determined by the abundance of the *aadA* amplicon normalized to the abundance of the *18S rDNA*. Relative quantification was performed using the ΔΔCt method^68^.

### Modelling and statistics

The quantitative effects of genotype and stress treatments on paternal plastid transmission, plastid inclusion in the generative cell and pollen survival (after chilling stress, or when comparing WT^ptGFP^ and *dpd1*^ptGFP^ mutant) were estimated using generalized linear models. Models were constructed with the maximum-likelihood method in R v3.5.3 (https://www.R-project.org/). Genotype, treatment and experiment (stage of visualization in pollen; independent experiments to determine plastid transmission) were proposed a priori as explanatory variables. Five models were selected for data analysis (Models 1–5).

The datasets for plastid inclusion in the generative cell (for Model 2), pollen survival under chilling stress (Model 4) and pollen survival in the wild-type and the *dpd1* genotypes (Model 5) were modelled as proportions of binary outcomes using the binomial distribution. The total amount of pollen grains analysed per unit of replication was provided in the ‘weights’ argument of glm(), as recommended^69^. The two paternal plastid transmission datasets (one representing Experiment 1, the other including Experiments 1, 2 and 3) were modelled using the negative binomial distribution (for Models 1 and 3), after evidence of overdispersion was found in preliminary Poisson versions of these models. The unit of replication in Experiments 1, 2 and 3 is the ‘harvest’: a batch of seeds obtained from a set of maternal recipients (5–30 plants) fertilized by pollen donors from a single treatment (3–8 plants). For modelling of proportions and rates, the count of seedlings with sectors per unit of replication was set as the response variable, while the total amount of seedlings analysed was provided as offset. ‘log’ was used as the link function for all models.

For each dataset, a starting model was generated that includes effect parameters for proposed explanatory variables and interactions. All effect parameters were modelled relative to specific levels of the explanatory variable (‘contrast to treatment’). Models were selected according to minimal AIC (Akaike’s Information Criterion), as they are considered the most parsimonious^70^: a version corrected for small sample sizes (AICc) was used as recommended previously^71^. From the starting and following models, parameters were removed sequentially and only if they led to a reduction in AICc. Overdispersion of the preliminary Poisson models was checked by performing a likelihood ratio test (LRT): this change resulted in a large reduction in AICc. The final models with minimal AICc (one per dataset) were designated Models 1–5. Goodness of fit was tested for these models using LRTs comparing (1) each selected model against a ‘saturated’ model, where the test provides a general measure of whether the model fits the data well, or (2) the selected model against a competing model to measure relative goodness of fit. Models 1–5 were confirmed to fit as well as the saturated model. Parameter estimates in the selected models were evaluated with simultaneous Wald’s *z*-tests (two-tailed). No corrections for multiple comparisons were applied for *z*-tests.

Negative binomial models were constructed using the glm.nb() function of MASS package v7.3.54^72^. Parameter values, standard errors and Wald *z*-statistics with *P* values were retrieved using the insight package v0.2.0 (https://easystats.github.io/insight/), or directly from the glm() and glm.nb() outputs (Extended Data Table 2). Parameters, errors and CI95s (95% confidence intervals) were transformed for the models to be log-linear in base 10. AICc were obtained through the MuMIn package v1.43.6 (https://CRAN.R-project.org/package=MuMIn). Wald CI95s for parameter estimates were calculated using the confint.default() function in the base package. Means fitted by the model were obtained from the glm() output, and CI95s for the estimated means were obtained by using the ciTools package v0.6.1 (https://CRAN.R-project.org/package=ciTools). LRTs for overdispersion were calculated using odTest() from the pscl package v1.5.5 (https://github.com/atahk/pscl/), and the remaining LRTs were performed using the base stats package. R was accessed through R Studio v2022.07.2 Build 576.

### Reporting summary

Further information on research design is available in the Nature Portfolio Reporting Summary linked to this article.

### Supplementary information

Reporting Summary

Supplementary Video 1 Three-dimensional confocal laser-scanning imaging of early binucleate pollen developing under standard greenhouse conditions. Early binucleate pollen was harvested from transplastomic WT^ptDsRed^ plants grown under standard conditions, followed by staining with aniline blue. The callose cell wall (grey) separating the vegetative cell and the newly formed generative cell was visualized by *z*-stack confocal imaging with optimized settings and a step size of ~0.34 µm (width of the slices: 8.5 µm). No plastids (magenta) are observed within the generative cell, in line with efficient exclusion of paternal plastids from the generative cell during male gametogenesis. This experiment was repeated four times and a representative video is shown.

Supplementary Video 2 Three-dimensional confocal imaging of early binucleate pollen developing under chilling stress. Early binucleate pollen were harvested from transplastomic WT^ptDsRed^ plants grown under chilling stress (10 °C), followed by staining with aniline blue. The callose cell wall (grey) separating the vegetative cell and the newly formed generative cell was visualized by *z*-stack confocal imaging with optimized settings and a step size of ~0.34 µm (width of the slices: 11 µm). A paternal plastid (magenta) is observed within the generative cell, indicating that the plastid exclusion mechanism during male gametogenesis is compromised at low temperature. This experiment was repeated four times and a representative video is shown.

Supplementary Video 3 Time-lapse confocal imaging of early pollen tube development during in vitro germination under chilling stress. Mature pollen grains were harvested from transplastomic WT^ptDsRed^ plants grown under chilling stress (10 °C), followed by pollen germination in vitro and staining with SYTO11, a nucleic acid-binding dye. The generative cell nucleus (GCN) within the growing pollen tube is visualized by green SYTO11 fluorescence. Paternal plastids (arrowhead, magenta) are closely associated with the GCN and enclosed by the generative cell membrane. Consequently, these plastids display similar movement dynamics as the GCN. By contrast, plastids outside of the generative cell (asterisk) display a much more rapid movement due to intense cytoplasmic streaming in the growing tube. This experiment was repeated ten times and a representative video is shown.

### Source data

Source Data Fig. 1 Unprocessed agarose gel and southern blot for assembly of Fig. 1e. Left: agarose gel with markers. Right: samples of the gel transferred to the blot.

Source Data Fig. 2 Ct values and calculations for relative quantification of plastid DNA.

## Figures and Tables

**Extended Data Table 1:**
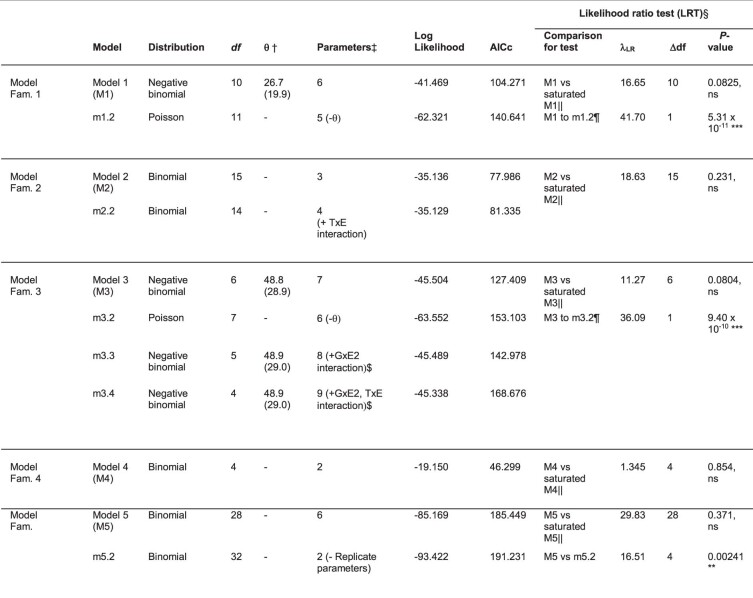
Specification of generalized linear models for analysis of paternal plastid transmission and plastid inclusion

Models 1–5 are the models analyzed in the manuscript. Models within a model family (Model Fam.) that are designated by a lowercase m are intermediates in the modelling process (that is, have parameters that were eliminated or added during the AICc minimization process). All models have ‘log’ as link function.

†Dispersion parameter θ estimated from the data. The standard error of the estimate is given in parentheses.

‡ Includes the intercept, parameters for the effects of variable, and interactions. In negative binomial models, it also includes the dispersion parameter θ. The extra parameters (+) or the missing parameters (-) that the respective model includes compared to the selected model are indicated in parentheses. T, Temperature; G, Genotype; E, Experiment (in the case of Model 2, E refers to the experiments performed at different stages of development, whereas in the case of Model 3, E refers to the three independent experiments conducted to determine the rate of paternal plastid transmission).

§LRT compares two models by checking whether λ_LR_ (difference in residual deviances) surpasses the critical value χ^2^_0.05_ with Δdf degrees of freedom, where Δdf is equivalent to the difference in the number of parameters between the two models. For all LRTs: *α* = 0.05; ns, *P* > 0.05; **, *P* < 0.01; ***, *P* < 0.001. Null hypotheses are formulated depending on the comparisons. LRTs are single-tailed by definition, and no corrections for multiple comparisons are performed.

|| LRT between a candidate model and the saturated model built from the same data (also called deviance test) is a general measure of goodness of fit of the candidate model. The saturated model has as many parameters as observations, and it fits maximally to the data by definition (residual deviance is zero). Thus, λ_LR_ is equal to the residual deviance of the candidate model. The null hypothesis is that the candidate model fits the data as well as the saturated model. Non-rejection of the null hypothesis is interpreted as evidence that the tested model fits well the data.

¶ LRT between a negative binomial model and its nested Poisson is a test for data overdispersion (when the conditional variance is higher than the conditional mean). The null hypothesis of the test is that the Poisson and negative binomial models fit to the data equally well. A rejected null hypothesis indicates the negative binomial model fits better to the data.

$ Model Family 3 includes the calculation of the interaction between genotype and temperature across all experiments, as well as the interactions involving Experiment 2. By contrast, triple interaction terms of the form GxTxE could not be calculated, as well as the GxE and TxE interactions involving Experiment 3. The independent experiments of paternal plastid transmission did not always contain experimental groups of all levels of genotype and temperature, thus leaving those interactions undefined for the dataset.

**Extended Data Table 2:**
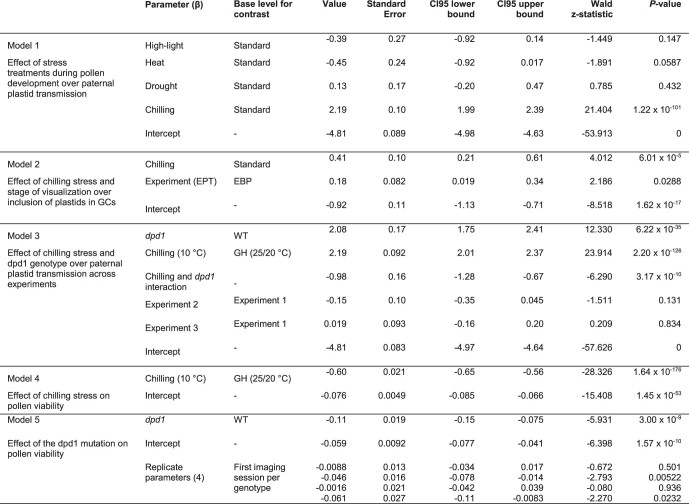
Parameter estimates obtained from modeling

Parameter values correspond to fold changes expressed in log_10_. Wald *z*-tests (*z*-statistic) are two-tailed with no corrections for multiple comparisons.

**Extended Data Table 3:**
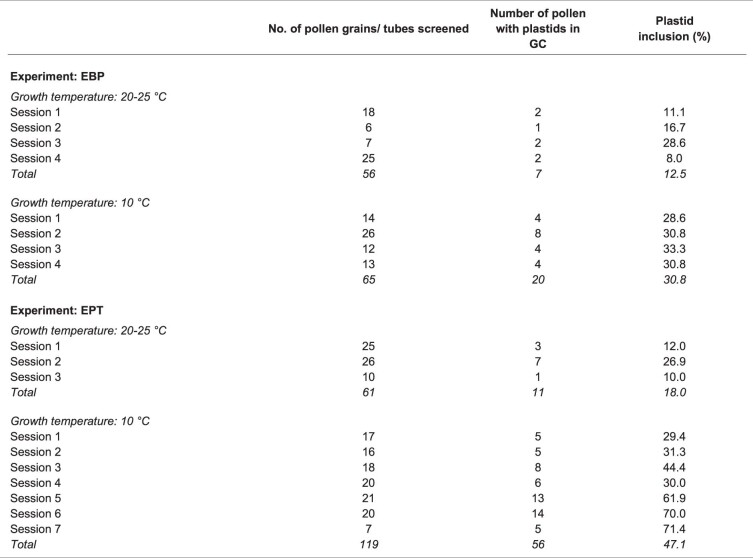
Proportions of plastid inclusion in the generative cell across imaging experiments

**Extended Data Table 4:**
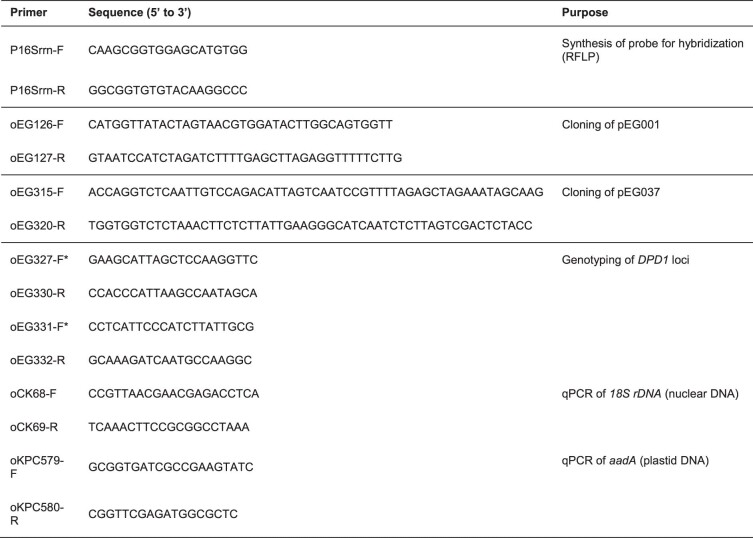
Synthetic oligonucleotides used in this study

* Primer used for Sanger sequencing (genotyping)

## Data Availability

Data supporting the findings of this work are available within the paper and its Supplementary Information files. Sequences from *Arabidopsis* (AT5G26940.1 and AT4G15440) are available through TAIR (https://www.arabidopsis.org/). Genomic sequences from *Nicotiana* (scaffolds Nitab4.5_0002715 and Nitab4.5_0014337) and transcripts (Nitab4.5_0002715g0070.1 and Nitab4.5_0014337g0020.1) are available at Sol Genomics Network (https://solgenomics.net/). Source data are provided with this paper.

## Code availability

The R code used for assembling the statistical models is available on GitHub at https://github.com/egonzalezduran/modelsplastidinheritance.

## References

[1] Hoekstra RF (2000). Evolutionary origin and consequences of uniparental mitochondrial inheritance. Hum. Reprod..

[2] Greiner S, Sobanski J, Bock R (2015). Why are most organelle genomes transmitted maternally?. BioEssays.

[3] Birky CW, Maruyama T, Fuerst P (1983). An approach to population and evolutionary genetic theory for genes in mitochondria and chloroplasts, and some results. Genetics.

[4] Birky CW (2008). Uniparental inheritance of organelle genes. Curr. Biol..

[5] Havird JC, Hall MD, Dowling DK (2015). The evolution of sex: a new hypothesis based on mitochondrial mutational erosion. BioEssays.

[6] Muller HJ (1964). The relation of recombination to mutational advance. Mutat. Res..

[7] Blanchard JL, Lynch M (2000). Organellar genes - why do they end up in the nucleus?. Trends Genet..

[8] Khakhlova O, Bock R (2006). Elimination of deleterious mutations in plastid genomes by gene conversion. Plant J..

[9] Smith JM, Haigh J (1974). The hitch-hiking effect of a favourable gene. Genet. Res..

[10] Wolfe KH, Li W-H, Sharp PM (1987). Rates of nucleotide substitutions vary greatly among plant mitochondrial, chloroplast, and nuclear DNAs. Proc. Natl Acad. Sci. USA.

[11] Drouin G, Daoud H, Xia J (2008). Relative rates of synonymous substitutions in the mitochondrial, chloroplast and nuclear genomes of seed plants. Mol. Phylogenet. Evol..

[12] Wu Z, Waneka G, Broz AK, King CR, Sloan DB (2020). MSH1 is required for maintenance of the low mutation rates in plant mitochondrial and plastid genomes. Proc. Natl Acad. Sci. USA.

[13] Cho Y, Mower JP, Qiu Y-L, Palmer JD (2004). Mitochondrial substitution rates are extraordinarily elevated and variable in a genus of flowering plants. Proc. Natl Acad. Sci. USA.

[14] Guisinger MM, Kuehl JV, Boore JL, Jansen RK (2008). Genome-wide analyses of Geraniaceae plastid DNA reveal unprecedented patterns of increased nucleotide substitutions. Proc. Natl Acad. Sci. USA.

[15] Parkinson CL (2005). Multiple major increases and decreases in mitochondrial substitution rates in the plant family Geraniaceae. BMC Evol. Biol..

[16] Sloan DB (2012). Rapid evolution of enormous, multichromosomal genomes in flowering plant mitochondria with exceptionally high mutation rates. PLoS Biol..

[17] Medgyesy P, Fejes E, Maliga P (1985). Interspecific chloroplast recombination in a *Nicotiana* somatic hybrid. Proc. Natl Acad. Sci. USA.

[18] Thanh ND, Medgyesy P (1989). Limited chloroplast gene transfer via recombination overcomes plastome-genome incompatibility between *Nicotiana tabacum* and *Solanum tuberosum*. Plant Mol. Biol..

[19] Baldev A (1998). Recombination between chloroplast genomes of *Trachystoma ballii* and *Brassica juncea* following protoplast fusion. Mol. Gen. Genet..

[20] Barnard-Kubow KB, McCoy MA, Galloway LF (2017). Biparental chloroplast inheritance leads to rescue from cytonuclear incompatibility. New Phytol..

[21] Tsitrone A, Kirkpatrick M, Levin DA (2003). A model for chloroplast capture. Evolution.

[22] Acosta MC, Premoli AC (2010). Evidence of chloroplast capture in South American *Nothofagus* (subgenus *Nothofagus*, Nothofagaceae). Mol. Phylogenet. Evol..

[23] Roux F (2016). Cytonuclear interactions affect adaptive traits of the annual plant *Arabidopsis thaliana* in the field. Proc. Natl Acad. Sci. USA.

[24] Bock DG, Andrew RL, Rieseberg LH (2014). On the adaptive value of cytoplasmic genomes in plants. Mol. Ecol..

[25] Zouros E, Oberhauser Ball A, Saavedra C, Freeman KR (1994). An unusual type of mitochondrial DNA inheritance in the blue mussel *Mytilus*. Proc. Natl Acad. Sci. USA.

[26] Shi L, Zhu T, Mogensen HL, Smith SE (1991). Paternal plastid inheritance in alfalfa: plastid nucleoid number within generative cells correlates poorly with plastid number and male plastid transmission strength. Curr. Genet..

[27] Metzlaff M, Börner T, Hagemann R (1981). Variations of chloroplast DNAs in the genus *Pelargonium* and their biparental inheritance. Theor. Appl. Genet..

[28] Szmidt AE, Alden T, Hällgren J-E (1987). Paternal inheritance of chloroplast DNA in *Larix*. Plant Mol. Biol..

[29] Mogensen HL (1996). The hows and whys of cytoplasmic inheritance in seed plants. Am. J. Bot..

[30] Matsushima R, Hu Y, Toyoda K, Sakamoto S, Sakamoto W (2008). The model plant *Medicago truncatula* exhibits biparental plastid inheritance. Plant Cell Physiol..

[31] Ruf S, Karcher D, Bock R (2007). Determining the transgene containment level provided by chloroplast transformation. Proc. Natl Acad. Sci. USA.

[32] Azhagiri AK, Maliga P (2007). Exceptional paternal inheritance of plastids in *Arabidopsis* suggests that low-frequency leakage of plastids via pollen may be universal in plants. Plant J..

[33] Hagemann R, Schröder M-B (1989). The cytological basis of the plastid inheritance in angiosperms. Protoplasma.

[34] Zhang Q, Liu Y (2003). Examination of the cytoplasmic DNA in male reproductive cells to determine the potential for cytoplasmic inheritance in 295 angiosperm species. Plant Cell Physiol..

[35] Zhou Q (2016). Mitochondrial endonuclease G mediates breakdown of paternal mitochondria upon fertilization. Science.

[36] Tilney-Bassett RAE (1994). Nuclear control of chloroplast inheritance in higher plants. J. Heredity.

[37] Svab Z, Maliga P (2007). Exceptional transmission of plastids and mitochondria from the transplastomic pollen parent and its impact on transgene containment. Proc. Natl Acad. Sci. USA.

[38] Corriveau JL, Goff LJ, Coleman AW (1990). Plastid DNA is not detectable in the male gametes and pollen tubes of an angiosperm (*Antirrhinum majus*) that is maternal for plastid inheritance. Curr. Genet..

[39] Svab Z, Maliga P (1991). Mutation proximal to the tRNA binding region of the *Nicotiana* plastid 16S rRNA confers resistance to spectinomycin. Mol. Gen. Genet..

[40] Ahlert D, Ruf S, Bock R (2003). Plastid protein synthesis is required for plant development in tobacco. Proc. Natl Acad. Sci. USA.

[41] Matsushima R (2011). A conserved, Mg2+-dependent exonuclease degrades organelle DNA during *Arabidopsis* pollen development. Plant Cell.

[42] Takami T (2018). Organelle DNA degradation contributes to the efficient use of phosphate in seed plants. Nat. Plants.

[43] Greiner S, Bock R (2013). Tuning a ménage à trois: co-evolution and co-adaptation of nuclear and organellar genomes in plants. BioEssays.

[44] Zupok A (2021). A photosynthesis operon in the chloroplast genome drives speciation in evening primroses. Plant Cell.

[45] De Storme N, Copenhaver GP, Geelen D (2012). Production of diploid male gametes in *Arabidopsis* by cold-induced destabilization of postmeiotic radial microtubule arrays. Plant Physiol..

[46] Birky CW (1995). Uniparental inheritance of mitochondrial and chloroplast genes: mechanisms and evolution. Proc. Natl Acad. Sci. USA.

[47] Wada M, Suetsugu N (2004). Plant organelle positioning. Curr. Opin. Plant Biol..

[48] Wada M, Kong S-G (2018). Actin-mediated movement of chloroplasts. J. Cell Sci..

[49] Wang X, Sheng X, Tian X, Zhang Y, Li Y (2020). Organelle movement and apical accumulation of secretory vesicles in pollen tubes of *Arabidopsis thaliana* depend on class XI myosins. Plant J..

[50] Moison M (2010). Cytoplasmic phylogeny and evidence of cyto-nuclear co-adaptation in *Arabidopsis thaliana*. Plant J..

[51] Boussardon C (2019). Novel cytonuclear combinations modify *Arabidopsis thaliana* seed physiology and vigor. Front. Plant Sci..

[52] Jaramillo-Correa JP, Bousquet J (2005). Mitochondrial genome recombination in the zone of contact between two hybridizing conifers. Genetics.

[53] Apitz J, Weihe A, Pohlheim F, Börner T (2013). Biparental inheritance of organelles in *Pelargonium*: evidence for intergenomic recombination of mitochondrial DNA. Planta.

[54] Alwadani KG, Janes JK, Andrew RL (2019). Chloroplast genome analysis of box-ironbark *Eucalyptus*. Mol. Phylogenet. Evol..

[55] Menczel L, Morgan A, Brown S, Maliga P (1987). Fusion-mediated combination of Ogura-type cytoplasmic male sterility with *Brassica napus* plastids using X-irradiated CMS protoplasts. Plant Cell Rep..

[56] Chung S-M, Gordon VS, Staub JE (2007). Sequencing cucumber (*Cucumis sativus* L.) chloroplast genomes identifies differences between chilling-tolerant and -susceptible cucumber lines. Genome.

[57] Hertle, A. P., Haberl, B. & Bock, R. Horizontal genome transfer by cell-to-cell travel of whole organelles. *Sci. Adv*. **7**, eabd8215 (2021). 10.1126/sciadv.abd8215 PMC7775762 33523859

[58] Murashige T, Skoog F (1962). A revised medium for rapid growth and bio assays with tobacco tissue culture. Physiol. Plant..

[59] Bock R (2001). Transgenic plastids in basic research and plant biotechnology. J. Mol. Biol..

[60] Bock R (2015). Engineering plastid genomes: methods, tools, and applications in basic research and biotechnology. Annu. Rev. Plant Biol..

[61] Edwards KD (2017). A reference genome for *Nicotiana tabacum* enables map-based cloning of homeologous loci implicated in nitrogen utilization efficiency. BMC Genomics.

[62] Concordet J-P, Haeussler M (2018). CRISPOR: intuitive guide selection for CRISPR/Cas9 genome editing experiments and screens. Nucleic Acids Res..

[63] Ruf S (2019). High-efficiency generation of fertile transplastomic *Arabidopsis* plants. Nat. Plants.

[64] Coutu C (2007). pORE: a modular binary vector series suited for both monocot and dicot plant transformation. Transgenic Res..

[65] Gibson DG (2009). Enzymatic assembly of DNA molecules up to several hundred kilobases. Nat. Methods.

[66] Lampropoulos A (2013). GreenGate - a novel, versatile, and efficient cloning system for plant transgenesis. PLoS ONE.

[67] Doyle JJ, Doyle JL (1990). Isolation of plant DNA from fresh tissue. Focus.

[68] Livak KJ, Schmittgen TD (2001). Analysis of relative gene expression data using real-time quantitative PCR and the 2-ΔΔCT method. Methods.

[69] Dunn, P. K. & Smyth, G. K. in *Generalized Linear Models With Examples in R* (eds. DeVeaux, R., Fienberg, S.E & Olkin, I.) Ch. 9–10 (Springer, 2018).

[70] Bozdogan H (1987). Model selection and Akaike’s Information Criterion (AIC): the general theory and its analytical extensions. Psychometrika.

[71] Burnham KP, Anderson DR (2004). Multimodel inference: understanding AIC and BIC in model selection. Sociol. Meth. Res..

[72] Venables, W. N. & Ripley, B. D. *Modern Applied Statistics with S*. 4th edn (Springer, 2002).

