# Supplementary material for: Control of plastid inheritance by environmental and genetic factors
Source: Nat Plants. 2023 Jan 16;9(1):68–80. doi: 10.1038/s41477-022-01323-7 (PMC9873568; doi:10.1038/s41477-022-01323-7)
Supplement: Supplementary file 1 — Reporting Summary [file 41477_2022_1323_MOESM1_ESM.pdf]

nature portfolio

Corresponding author(s): Ralph Bock

Last updated by author(s): Nov 18, 2022

Reporting Summary

Nature Portfolio wishes to improve the reproducibility of the work that we publish. This form provides structure for consistency and transparency in reporting. For further information on Nature Portfolio policies, see our Editorial Policies and the Editorial Policy Checklist.

Statistics

For all statistical analyses, confirm that the following items are present in the figure legend, table legend, main text, or Methods section.

n/a	Confirmed	
☐	☒	The exact sample size (*n*) for each experimental group/condition, given as a discrete number and unit of measurement
☐	☒	A statement on whether measurements were taken from distinct samples or whether the same sample was measured repeatedly
☐	☒	The statistical test(s) used AND whether they are one- or two-sided<br>*Only common tests should be described solely by name; describe more complex techniques in the Methods section.*
☐	☒	A description of all covariates tested
☐	☒	A description of any assumptions or corrections, such as tests of normality and adjustment for multiple comparisons
☐	☒	A full description of the statistical parameters including central tendency (e.g. means) or other basic estimates (e.g. regression coefficient) AND variation (e.g. standard deviation) or associated estimates of uncertainty (e.g. confidence intervals)
☐	☒	For null hypothesis testing, the test statistic (e.g. *F*, *t*, *r*) with confidence intervals, effect sizes, degrees of freedom and *P* value noted<br>*Give P values as exact values whenever suitable.*
☒	☐	For Bayesian analysis, information on the choice of priors and Markov chain Monte Carlo settings
☒	☐	For hierarchical and complex designs, identification of the appropriate level for tests and full reporting of outcomes
☒	☐	Estimates of effect sizes (e.g. Cohen's *d*, Pearson's *r*), indicating how they were calculated

Our web collection on statistics for biologists contains articles on many of the points above.

Software and code

Policy information about availability of computer code

Data collection	No code was used to collect the data in this study.
Data analysis	Custom code was used for the assembly of generalized linear models and their hypothesis testing, using the R software v.3.5.3 and R Studio 2022.07.2 Build 576 and publicly available packages. A detailed explanation of the process is provided in the Methods section. The R packages used are MASS v.7.3-51.1, dplyr v.0.8.0.1, ggplot2 v.3.1.1, ciTools v. 0.5.1, insight v.0.2.0, pscl v. 1.5.2, MuMIn v. 1.43.6, rstudioapi v. 0.10. The code has been deposited in GitHub, accessible through https://github.com/egonzalezduran/modelsplastidinheritance

For manuscripts utilizing custom algorithms or software that are central to the research but not yet described in published literature, software must be made available to editors and reviewers. We strongly encourage code deposition in a community repository (e.g. GitHub). See the Nature Portfolio guidelines for submitting code & software for further information.

Data

Policy information about availability of data

All manuscripts must include a data availability statement. This statement should provide the following information, where applicable:

- Accession codes, unique identifiers, or web links for publicly available datasets
- A description of any restrictions on data availability
- For clinical datasets or third party data, please ensure that the statement adheres to our policy

All data supporting the findings of this study are available within the paper (and supplementary information files). Source data are provided with this manuscript.

Sequences from Arabidopsis (AT5G26940.1 and AT4G15440) are available through TAIR (https://www.arabidopsis.org/). Genomic sequences from Nicotiana (scaffolds Nitab4.5_0002715 and Nitab4.5_0014337) and transcripts (Nitab4.5_0002715g0070.1 and Nitab4.5_0014337g0020.1) are available at Sol Genomics Network (https://solgenomics.net/)

Human research participants

Policy information about studies involving human research participants and Sex and Gender in Research.

Reporting on sex and gender	N/A
Population characteristics	N/A
Recruitment	N/A
Ethics oversight	N/A

Note that full information on the approval of the study protocol must also be provided in the manuscript.

Field-specific reporting

Please select the one below that is the best fit for your research. If you are not sure, read the appropriate sections before making your selection.

☒ Life sciences ☐ Behavioural & social sciences ☐ Ecological, evolutionary & environmental sciences

For a reference copy of the document with all sections, see nature.com/documents/nr-reporting-summary-flat.pdf

Life sciences study design

All studies must disclose on these points even when the disclosure is negative.

Sample size	Sample sizes were not pre-determined. In paternal plastid transmission experiments, Experiment 1 was exploratory, and there was no prior expectation as to how large the effect of treatments on plastid transmission could be. After it became evident that chilling stress largely increased paternal transmission, the number of crosses performed was reduced in subsequent experiments. Experiment 2 was also exploratory (to determine the effect of the dpd1 mutation), but it became evident that the effects on plastid inheritance were very large. Experiment 3 was conducted to replicate the dpd1 finding: its sample size was set to be similar to that of Experiment 2.<br>In pollen imaging experiments, data collection was laborious and time consuming (due to the requirement for staining of the callose wall, the rarity of morphologically normal pollen grains, and the necessity of having them in the right plane for imaging), and thus limited sample sizes. Data were collected until it became clear that plastid inclusion in pollen from both stages showed a consistent trend.
Data exclusions	No datasets were excluded.
Replication	All findings in this manuscript have been successfully replicated and at least two independent experiments have been performed. Information units of replication are provided in the manuscript (see figure legends).
Randomization	Plants were assigned randomly to a treatment when relevant. For crosses, plants acting as maternal parents were assigned randomly to a group receiving pollen from paternal parent plants in a single treatment.
Blinding	Data collection and analysis was not blinded. There was no subjective parameter measured that would have benefited from a blinded design.

Reporting for specific materials, systems and methods

We require information from authors about some types of materials, experimental systems and methods used in many studies. Here, indicate whether each material, system or method listed is relevant to your study. If you are not sure if a list item applies to your research, read the appropriate section before selecting a response.

Materials & experimental systems

n/a	Involved in the study	
☒	☐	Antibodies
☒	☐	Eukaryotic cell lines
☒	☐	Palaeontology and archaeology
☒	☐	Animals and other organisms
☒	☐	Clinical data
☒	☐	Dual use research of concern

Methods

n/a	Involved in the study	
☒	☐	ChIP-seq
☒	☐	Flow cytometry
☒	☐	MRI-based neuroimaging
