# Supplementary figures and images for: Control of plastid inheritance by environmental and genetic factors
Source: Nat Plants. 2023 Jan 16;9(1):68–80. doi: 10.1038/s41477-022-01323-7 (PMC9873568; doi:10.1038/s41477-022-01323-7)

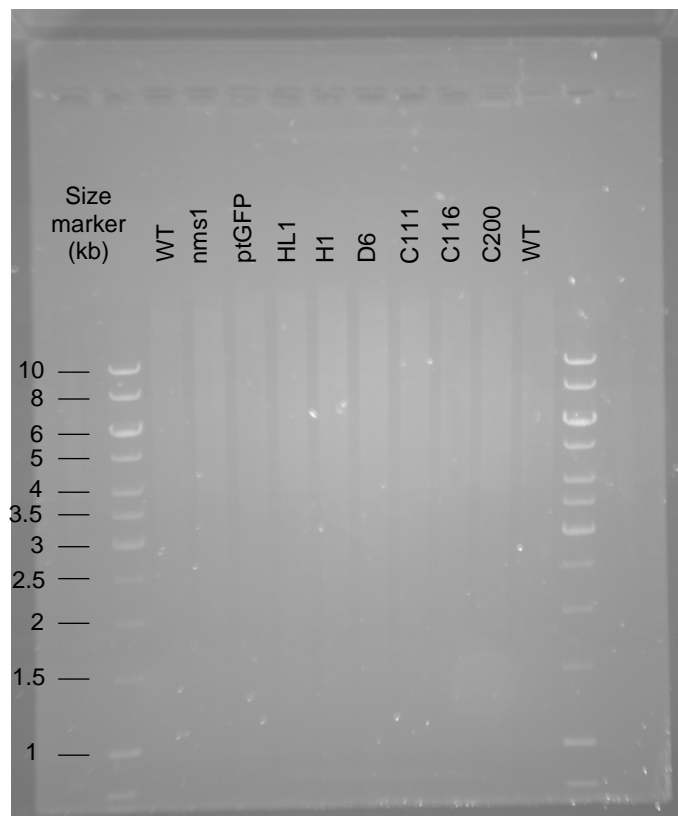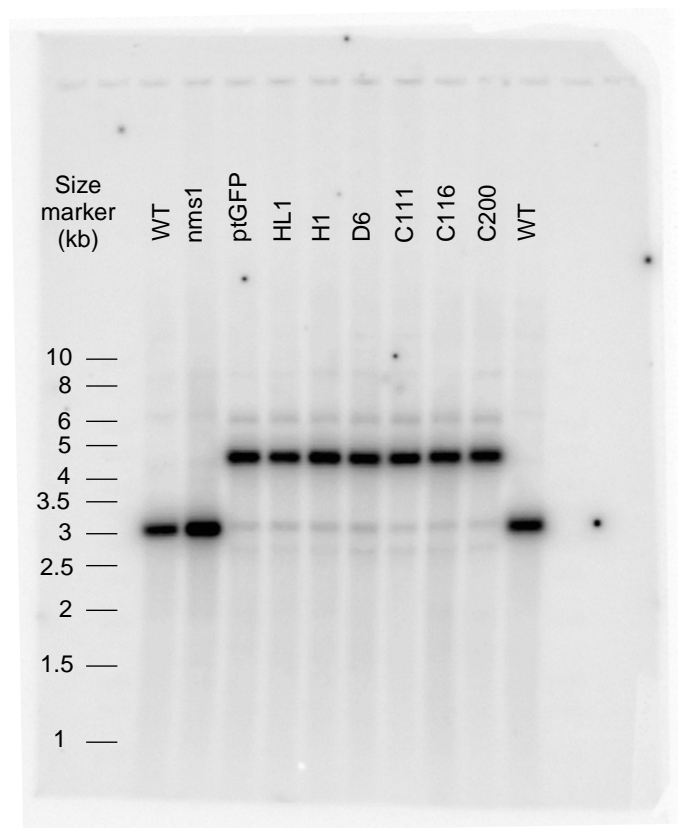

Supplement: Supplementary file 5 — Unprocessed agarose gel and southern blot for assembly of Fig. 1e. Left: agarose gel with markers. Right: samples of the gel transferred to the blot. [file 41477_2022_1323_MOESM5_ESM.pdf]
